# Recent Advances in Computer-Aided Drug Design and Drug Discovery

**DOI:** 10.3390/molecules31101606

**Published:** 2026-05-11

**Authors:** John Z. H. Zhang

**Affiliations:** Faculty of Synthetic Biology, Shenzhen University of Advanced Technology, Shenzhen 518055, China; zhzhang@phy.ecnu.edu.cn

The field of Computer-Aided Drug Design (CADD) has undergone remarkable transformations, evolving from a niche discipline into a cornerstone of modern pharmaceutical research [1]. As computational power advances and artificial intelligence (AI) rapidly mature, CADD is revolutionizing how we discover and develop new therapeutics. The first edition of this *Molecules* Special Issue, “Recent Advances in Computer-Aided Drug Design and Drug Discovery,” showcases this dynamic momentum. The collection of articles presented here captures important progress across structure-based and ligand-based approaches, AI-driven generative chemistry, and beyond—each contribution thoughtfully addressing both time-honored challenges and newly emerging frontiers.

The progress chronicled in this compilation is impressive. The integration of AI and machine learning (ML) has become the central driver of innovation in the field [2], enabling ultra-large-scale virtual screening and deep generative molecular design for unprecedented chemical space exploration [3,4]. In parallel, the revolutionary development of tools like AlphaFold has extended protein structure prediction beyond isolated proteins to the prediction of biomolecular complexes [5], dramatically expanding the scope of rational target selection. The maturation of predictive models for Absorption, Distribution, Metabolism, Excretion, and Toxicity (ADMET) properties has also resulted in more robust, interpretable, and accurate assessments of drug-likeness [1]. Collectively, these advances are not merely incremental; they are redefining what is computationally possible in the realm of drug discovery.

Yet, for all this promise, profound gaps remain. While AI has demonstrably accelerated the early stages of drug development, the translation of computational predictions into clinically approved therapies remains elusive. Persistent challenges include the difficulty of ensuring synthetic feasibility for AI-generated molecules [6,7], the pervasive issues of inconsistent experimental data quality [4,7], the often-insufficient generalizability of models across diverse chemical scaffolds [8], and the persistent “black-box” nature of many advanced AI systems [6]. These obstacles continue to hinder the reliability, clinical translatability, and real-world impact of many computational approaches.

It is precisely at the intersection of these rapid advances and persistent knowledge gaps that this Special Issue makes its most important contribution. The included articles directly confront the field’s most pressing challenges. They rigorously interrogate the limitations of current methodologies, exposing areas such as data quality and bias, model interpretability, and the often-overlooked gap between in silico predictions and experimental outcomes. By critically assessing where—and why—current approaches fail, this Special Issue provides the community with a necessary roadmap for improvement.

Looking forward, several future directions stand out as particularly promising. The emergence of “agentic” AI systems, which can autonomously perform functions including synthesis planning and molecular design, is beginning to reshape the R&D landscape [7]. The growing convergence of AI models with automated synthesis and high-throughput experimentation points toward closed-loop discovery systems that can learn iteratively from wet-lab feedback [7]. Furthermore, the pressing need to move beyond small molecules toward biologics, peptides, and macrocycles is opening new frontiers for ADMET prediction and structure-based design [8,9]. However, for these advances to achieve their full potential, the community must also confront foundational issues: investing in higher-quality shared datasets, developing explainable AI frameworks that can illuminate decision-making [7], and fostering truly interdisciplinary collaboration. The regulatory landscape is also evolving, with agencies like the FDA beginning to establish frameworks for AI’s role in drug development toward the end of the decade—a development that will demand careful navigation.
